# Hydrosudopathia

**Published:** 1838

**Authors:** Louis Fleury

**Affiliations:** Resident Surgeon of the Hospitals, member of the Anatomical Society, &c.


### II.—ANALYTICAL.

### Art. I.—Hydrosudopathia.

Hydrosudopathia, *or a Therapeutic system founded on the combined action of cold water and exciting cutaneous perspiration; by Louis Fleury, resident Surgeon of the Hospitals, member of the Anatomical Society, &c.,* [*Translated and abridged from the French.*]

Looking over one of a late number of the French Journals, (*Archives Generales de Medecine,*) we were struck with a new word, Hydrosudopathia, noticed by the Germans as, *Die Wassercuren des Priesnitz.* The heading of the article reminded us of Dr. Sangrado, whom we little dreamt of seeing revived and really acted out at the present day.

However, on a careful perusal, the importance which the subject has assumed, and the pleasant style of M. Fleury, who appears to be a young man of some spirit, induced the belief that an account of this new and astonishing mode of treatment would prove interesting to the readers of the Western Journal, and we accordingly offer the article, with the exception of the cases, as follows:

In a fertile valley of the Mountains of Austrian Silesia, between Glatz and Neiss, at an elevation of eighteen hundred feet above the Sea; there is a little hamlet, of seventeen families. But a few years since, the artist, or the traveller who loved picturesque scenery, was the only one who ever claimed the open hospitality of this colony of Shepherds and Husbandmen. Their very name was scarcely known in the town, six hundred feet below, at the foot of the cliff which supported their cabins—now the same town Freiwaldau, is but the resting place: while Græfenberg is the resort of German princes, and travellers from all parts of the world; who desert Carlsbad, Ems, and Baden!—Its name is on every tongue, the poets immortalize it in their verse, and the easy enthusiasm of our German neighbours has induced them to write several works descriptive of it.

This rapidly acquired renown is the work of a simple peasant, who was led by chance, perhaps as much as by observation, to found a Medical establishment, in which the mode of treatment will no doubt appear astonishing, since it depends upon the free administration of two powerful therapeutic agents, which are too much despised now-a-days; cold water, and exciting cutaneous perspiration.

Some details gathered from German works, and the notes furnished me by Baron D Ch———, who has studied the treatment upon the spot, may be read with interest.

Vincent Priesnitz was born the 4th of July 1799, in one of the cottages on the summit of the Græfenberg, and, as his parents were in easy circumstances, he received a pretty good education, which excited a spirit of observation, with uncommon tact and penetration; when he had scarcely grown up, he noticed, while assisting his father in rural labors, that in sprains, bruises and tumours of horses' feet, the cure was much hastened by bathing with cold water; having frequently verified this fact, and assured himself of its efficacy, he employed it on several animals: success crowned all his efforts, and he was inspired with great confidence in the virtues of cold water.

In 1816, the young Priesnitz was thrown from a fiery horse, that trod upon his face, severely bruised his left arm, and broke two of his ribs. A surgeon was called, who, having unsuccessfully attempted to reduce the fracture, declared, that if the patient escaped the danger which threatened him, he would be long disabled. The young man, dissatisfied with this opinion, determined to treat himself; for this end, he rested his chest against a chair, and taking an inspiration made the fragments resume their natural position; applied a wet towel as a bandage, drank cold water, and was well in a few days.

This simple cure struck the imagination of Priesnitz, he attributed to the means he had employed, what we see every day resulting from the unaided efforts of nature, and devoted himself with new ardour to investigating the general effects of cold, and the laws which regulate its application in the diseases of man. I will repeat here a single experiment: Two hogs were fed, one with cold, the other with hot food, in the first, the intestines were found firm, white and resisting, while in the second, they were reddened and soft, and so easily torn that they could not be used by the sausage maker.

Priesnitz, having recognized the good effects of cold water in the treatment of many diseases, soon thought that an indispensable condition to render its application as efficacious as possible, was to cause profuse and frequent transpiration of the skin, and the combination of these two means became the basis of his medication. He cured some cases of gout and rheumatism in this way; which were noised about the country and his house was soon too small to contain the numbers who came for advice. His reputation increased rapidly, and the Mountaineers soon regarded him as a protégé of Heaven: according to them, the water possessed no virtue in itself, and owed its action to the secret power of Priesnitz: it is thus every where, in the eyes of the vulgar, the simplest things assume a marvellous appearance without which they would often be rejected with disdain. But this very success made Priesnitz many enemies; the priests cast anathemas upon his devilish art: the physicians and veterinaries denounced him for practicing illegally, and the authorities were obliged to intervene.

In 1830, the Austrian government granted Priesnitz authority to receive patients and treat them in his own way: from this time his establishment was rapidly increased, for counting only fifty-four patients the first year, he had sixty-four the next, one hundred and eighteen in 1832, two hundred and six in 1833, two hundred and fifty-six in 1834, three hundred and forty-two in 1835, and four hundred and sixty-nine in 1836.

He has now built three large houses which cannot contain the patients who come from all parts, and the little town of Friewaldau has been obliged to erect a resting house *(Succursale)*.—Analogous establishments are formed elsewhere; Dr. Emel has organized one two leagues from Vienna, at Kaltenleitgeb, near Rodaun; Dr. Neiderfuhe, in the earldom of Glatz; Dr. Lehman, three miles from Breslau; it is said that others will be instituted in Bavaria, in Saxony, at Freiberg, in Wirtemberg and even at St. Petersburg.

The treatment which has gained so great reputation in Germany, consists of diet, cutaneous excitation, and cold water.

*Dietetic Regimen.*—According to Mr. Priesnitz, hot food produces injurious effects upon all animals; as man is subject to nearly the same general laws, he thinks that the frequent derangements of the alimentary canal, are entirely owing to our use of food at high temperatures, and that it will be easy to prevent, or remedy them by using it cold and by choosing water for our only drink. Another principle to which he attaches great importance, is, that dieting, instead of being useful, must generally enfeeble the patient, and deprive the economy of the power it so needs to combat disease; an opinion held by many others. This is the regimen at Græfenberg:—

At breakfast, cold milk, bread and butter;—any thing may be eaten at dinner except spices, and as much as the appetite demands; warm meats are allowed those who are slightly affected, but in serious cases, all the food is cold, cold water the only drink without exception—Supper is like the dinner. During the day, between meals cold water is to be drank frequently, from 20 to 30 glasses in twenty-four hours.

*Cutaneous Excitation.*—It has always been thought serviceable to excite frequent perspiration, as is proved by the *sudatori* of the Romans, Greeks, Turks, Russians, &c.;—but it is very important to know how to excite, to regulate and to terminate it in the most advantageous manner. Mr. Priesnitz thinks that all passive transpiration is hurtful; that steam is injurious to the lungs and brain; that a salutary transpiration must be produced by exercising and concentrating the vital functions, and he employs the following method :—

The patient goes to bed and is swathed with great care in a woollen covering ; one or two feather beds are super-added—the head is a little raised. When perspiration is well established, the windows are opened and the patient takes, the first day, a quarter glass of cold water ; which is insensibly augmented so that at the end of several days he takes a whole glass, every quarter of an hour. This is kept up according to the strength of the individual and the nature of the disease, from a quarter of an hour, to six and seven hours—counting from the moment when the perspiration is observed. At first, this state is not easily induced, but the sweat soon becomes so abundant, that Mr. D Ch—— has seen it soak through the covering and mattresses, and fall to the floor under the bed. When it is thought time to terminate the excretion, the feather beds are removed, the patient gets up without laying aside his covering, bathes his figure and chest with cold water, and plunges into a large basin of water ; which has been brought from the mountain in pipes ; its termperature never exeeeds 44 deg. of Fahrenheit, in winter it descends to 36 deg. and sometimes to 32 deg. They plunge into this basin at all seasons, at first, it is a mere immersion, afterward they remain two or three minutes, and sometimes longer. Priesnitz says he once remained in the bath ten hours, to cure a hot fever. Few have courage to encounter a sensation which is at first painful—such, terminate the perspiration in a bath with two or three inches of water at 59 or 68 deg. During their noviciate, which lasts about a week, the temperature is gradually reduced and the basin used. Immediately after the immersion, they dress, take a walk and return to breakfast.

*Application of cold water.*—Besides the baths of immersion, cold water is used partially, *loco dolenti*, and also in douches, enemata, and compresses.

*Hip bath.*—About three inches of cold water is poured into a suitable apparatus, in which the patient sits for twenty minutes or an hour, during which time all the parts are rubbed, and the *bas ventre* occasionally moistened.

*Foot Bath.*—The feet are placed in half an inch of cold water, rubbed one against the other ; or with the hand, and the water becomes rapidly warmed and sometimes absorbed.

*Bath for the limbs.*—The affected member is placed with a small quantity of water, in a vessel adapted to the shape of the parts—care is always taken to rub forcibly.

*Head bath.*—According to the seat of the disease, the face or the occiput is placed in a vessel containing three inches of water. It is always necessary to continue the process until the fluid be so warmed, at the expense of the caloric of the submerged part, as not to feel cold.

*Douches.*—Are of two sorts, those of the establishment, which are little used, and badly arranged; and those of the forest, situated in the mountains, half an hour's walk from the house, and unprotected; the water is brought in spouts, and is allowed to fall ten or fifteen feet in a column one inch and a half in diameter upon affected parts, not less than five minutes or more than one hour, care being taken to protect the head and chest. They are used at all seasons and temperature. Mr. D Ch—— saw persons exposed to them in the midst of snow and ice, and he took them himself, during last January and February.

*Enemata.*—Are used at night; at first they produce a painful sensation and a desire to reject them instantly, but the intestine becomes habituated to their presence.

*Wet Compresses*—Are applied to different parts according to the affection, frequently removed, or left in place until completely dry. Sometimes a girdle five feet long with eighteen inches at one end soaked in cold water, is applied around the abdomen.

*Wet cloths*—Are rolled around the patients like that used to excite perspiration. These are renewed every five minutes in acute inflammatory maladies, as pneumonia.

*Order of treatment, general effects, duration, and termination.*—The various curative means enumerated, are not indifferently employed; and it is impossihle to specify all the modifications to suit sex, age, strength and constitution of the patient, the kind of affection, its complicatoins, &c.; so that we shall speak in general terms.

At four o'clock in the morning the patient is woke up to be wrapped for a sweat, which is prolonged according to circumstances, this is terminated by the bath of immersion. Then a walk until breakfast, after an hour, a douche or hip bath, dine at noon --from three to six, a shorter sweat than in the morning, followed by the bath; sup at eight; at nine, a hip or foot bath or enema—retire at ten. Those who prefer but one sweat a day, prolong that of the morning until noon, after which they are free. Some concessions are made at first, but after a week all must submit to the rules just described. After some time cutaneous eruptions and boils make their appearance; diarrhœa comes on, abscesses form, or venereal symptoms reappear that had long lain dormant; some one of these phenomena is requisite to make the cure efficacious. This crisis is usually developed at the end of six weeks or two months, and may be renewed at intervals. The treatment is continued four or six months, or a year, according to the severity of the disease.

Though M. Priesnitz thinks he can successfully combat many diseases, he does not pretend to make his treatment a panacea, in which he shows more good sense than most inventors. He attributes great power to his method in rheumatism, gout, constitutional syphilis,diseases of the skin, even the exanthemata, in hæmorrhoids, fistula, caries, chronic engorgements of different tissues, scrofula, chronic affections of the digestive tube; he has recourse to it in some acute inflammatory diseases—as angina and opthalmia; he even ventures to envelope in the cold water cloth a patient with pneumonia or peritonitis.

After citing some wonderful cures, and taken from the German authors, or communicated to him, complaining of the very common want of accuracy in their diagnosis, M. Fleury, concludes by a separate examination of the means employed, the treatment as a whole, and its action upon different diseases.

*Internal use of cold water.*—Without repeating all the arguments that have been accumulated to prove that water is the *natural* drink of man, I will merely state that of all liquids, it best quenches thirst, that it facilitates digestion, and that according to the experience of M. M. Leuret and Lassaigne, it is indispensable to the formation of chyle.

M. Ratier says;—"that certain cures, due to water alone, are often attributed to something else. It diminishes the febrile heat, arouses the secretions and exhalations and evidently modifies their products. It were better for physicians to disseminate these truths than to prescribe with so much ado,—tisans, that have no other virtue. There are few diseases in which the proper administraion of water may not, at least aid the cure."

M. Guerard says, "if we more frequently obeyed the natural indications, pure water would be more used than any tisan."

All writers agree generally, with those cited, but in spite of such favorable opinions of the use of cold water, no one conceived or dared to make consecutive and complete experiments. The isolated facts recorded by Senac, Chomel, and others, have not excited the researches that they would have justified.

*External application.*—Water has been oftener and more methodically employed in this manner, but we find it alternately praised and rejected, from the time of Hippocrates who acknowledged its great power. It is now much used in surgery; the good effects obtained by M. M. Berard, Cloquet, Gerdy, Sanson, &c., are well known, and Percy assures us that applications of cold water may cure spontaneous luxations and incomplete anchyloses.

It has been less used *for internal affections.*—Van Sweiten relates that an Italian physician treated hæmoptisis successfully by applying compresses, soaked in cold water, to the naked chest.—A case was reported in this Journal, of the efficacy in puerperal peritonitis, of ice water, internally and externally—the English have used affusions at a temperature of 51 deg. in Scarlatina; Phytt cured obstinate constipation by affusions to the feet.

I will not dilate upon the proper temperature that should be given to the water; we know that cold acts in three ways; at very low temperatures it causes the death of the tissues in the same way as extreme heat—when moderate, it acts in different ways, thus, it is tonic or excitant,— by the reaction it produces when used for a short time, but sedative when long continued.

*Excitement of the skin.*—In arousing perspiration, the skin is first softened, and rendered fit to receive the action of therapeutic agents, the natural secretion is excited, and made to re-appear when suppressed. As it is a frequent *natural crisis*, it is often highly serviceable to induce it.

*The whole treatment.*—M. Priesnitz brings into play all the different properties ascribed to cold water and perspiration, and does not fear to unite them, though generally considered incompatible. We know the dangers attributed to the use of water when there is perspiration. The practice of the Russians coming out of their vapour baths to plunge into water or snow, is not explained by saying that their return to the stove would take away the danger of such a custom. Baglivi, Rush, and Berard, have reported cases of death following a draught of cold water taken during free perspiration. Priesnitz says that the cold water when a man is perspiring can only be dangerous when, from any cause, the respiratory apparatus is excited; on the contrary, when respiration is not accelerated it is salutary.

*Application of the treatment.*—We must here establish some distinction: in the case of *acute inflammatory affections*, cold water is employed as a sedative, that is, continuously. In Surgery we see great advantages arising from its use in this way, and it may be asked if a remedy by which we can suspend the circulation, as long as we choose, be not preferable to such means, as local or general bloodletting, that act by drawing off from the sanguine mass a small quantity, inappreciable to the affected organ, or a large amount that would be prejudicial to the whole economy? M. Forville regards cold affusions as the only remedy that will cut short meningitis, why are they not used in other inflammations? In the actual state of our notions, it certainly appears very strange to surround a pneumonic patient with a cold wet cloth, but should this motive induce us to proscribe a practice proved to be rapidly and happily efficacious?

*In chronic local diseases*, cold water may be used as a sedative or excitant, and since very good effects have been observed to follow its application in gastralgia and other neuroses, in engorgements, cold abscesses, &c., we admit that it may succeed in other cases.

*General chronic diseases.*—Here the question turns upon a doctrine, which though very ancient, is far from being determined, that of *crisis*. Priesnitz, who admits the doctrine, uses cold water as a tonic, to enable the economy to resist the disease, and to give the *natural healing power* energy to bring on the crisis. The excitement aroused in the skin, effects the double purpose of increasing the intensity of action of the cold water, and of directing the crisis to the skin and subcutaneous cellular tissue. So he does not consider the cure manifest until these phenomena appear, and he says they are never wanting when properly excited. He acknowledges, however numerous exceptions to this rule, in old age, and he has determined to receive into his establishment, no more patients advanced in years.

I will not further discuss this topic, in favour of which so many well observed facts may be advanced, in opposition to the negations of others—but it may be useful to act upon the suggestion of Bordeu, and *clear up these important questions by observation.* This will be especially necessary in regard to diseases that are not well understood, as cutaneous affections, syphilis, &c.

These views have induced me to publish an account of Priesnitz, whose method is certainly susceptible of modifications in form, but undoubtedly energetic, and already remarkable for its negative results, were there no others.

April 1838. J. A. W.

